# Optimal integration of kinematic and ball-flight information when perceiving the speed of a moving ball

**DOI:** 10.3389/fspor.2022.930295

**Published:** 2022-11-29

**Authors:** Hiroki Nakamoto, Kazunobu Fukuhara, Taiga Torii, Ryota Takamido, David L. Mann

**Affiliations:** ^1^Faculty of Physical Education, National Institute of Fitness and Sports in Kanoya, Kanoya, Japan; ^2^Department of Health Promotion Science, Graduate School of Human Health Science, Tokyo Metropolitan University, Tokyo, Japan; ^3^Research Into Artifacts Center, Center for Engineering, School of Engineering, University of Tokyo, Tokyo, Japan; ^4^Department of Human Movement Sciences, Amsterdam Movement Sciences and Institute of Brain and Behavior Amsterdam, Vrije Universiteit Amsterdam, Amsterdam, Netherlands

**Keywords:** baseball, speed perception, virtual reality, time to contact (TTC), sports expertise

## Abstract

In order to intercept a moving target such as a baseball with high spatio-temporal accuracy, the perception of the target's movement speed is important for estimating when and where the target will arrive. However, it is unclear what sources of information are used by a batter to estimate ball speed and how those sources of information are integrated to facilitate successful interception. In this study, we examined the degree to which kinematic and ball-flight information are integrated when estimating ball speed in baseball batting. Thirteen university level baseball batters performed a ball-speed evaluation task in a virtual environment where they were required to determine which of two comparison baseball pitches (i.e., a reference and comparison stimuli) they perceived to be faster. The reference and comparison stimuli had the same physical ball speed, but with different pitching movement speeds in the comparison stimuli. The task was performed under slow (125 km/h) and fast (145 km/h) ball-speed conditions. Results revealed that the perceived ball-speed was influenced by the movement speed of the pitcher's motion, with the influence of the pitcher's motion more pronounced in the fast ball-speed condition when ball-flight information was presumably less reliable. Moreover, exploratory analyses suggested that the more skilled batters were increasingly likely to integrate the two sources of information according to their relative reliability when making judgements of ball speed. The results provide important insights into how skilled performers may make judgements of speed and time to contact, and further enhance our understanding of how the ability to make those judgements might improve when developing expertise in hitting.

## Introduction

The interception of a moving creature or object is a fundamental action necessary for animals and humans to achieve their behavioral goals in a dynamic environment (e.g., hunting for prey and/or picking up a piece of sushi from a moving conveyer belt). Predicting when and where a moving object will arrive in the near future is critical to the success of these interceptive tasks. In particular, when extremely spatiotemporally accurate interception is required in a highly uncertain and time-constrained environment such as in sports (e.g., baseball hitting requires accuracy of <10 ms in time and 2 cm in space), the prediction accuracy is expected to have a direct impact on interceptive performance (1–4). Therefore, learning to intercept requires the performer to clarify what information to use and how to use it in order to achieve accurate temporal and spatial predictions.

In terms of predicting when an object will arrive (i.e., time-to-contact: TTC), early studies showed that a visual variable called tau could be the primary source of information for establishing time-to-contact (5–7). Specifically, the TTC can be calculated by dividing the visual angle of the object by the rate of change of it on the retina. Although this strategy enables an estimation of the TTC in the presence of a very short latency (6, 8), and has a neuronal basis (4, 9), it has been criticized for its inapplicability to a variety of real-life situations (10, 11). Alternatively, TTC could be estimated using information inherent in binocular disparity (12) and/or kinematic information such as the distance, speed, and acceleration of the target (10, 12–14). Above all, speed information is considered important not only for predicting when, but also for predicting where an object will arrive. For example, a study of baseball batting showed that the height at which the ball crossed the plate could be predicted indirectly from an estimate of the ball speed (1, 12). Thus, speed estimation is believed to be crucial for the spatio-temporal prediction of moving objects in natural interception. Indeed, it has been shown that swing timing and the height of the swing location are modulated on the basis of ball speed (12, 15, 16).

Although of course the information available from the ball itself is critical for the estimation of ball speed, it seems unlikely that the speed estimation necessary to achieve skilled interception would be obtained only from the ball information itself. Bahill and Karnavas (1) have argued that “the speed estimator probably uses memory and other sensory inputs: some visual, such as the motion of the pitcher's arms and body” (p. 8). Indeed a whole body of research on anticipation in sports has shown that skilled athletes have a superior capacity to pick-up information from an opponent's actions to make inferences about the action outcome (17, 18). From a baseball perspective, this means that skilled batters are better able to pick-up information from the pitcher's movements to predict the type of pitch and other ball-flight characteristics such as the height and direction of the pitch (2, 3, 19–21). From the viewpoint of ball speed, since angular velocities of various body parts such as the pitching arm are related to ball speed [e.g., (22, 23)], ball speed is indeed related to the speed of the pitching movement. Recently, Takamido et al. (24) examined this in a softball batting prediction task by manipulating the movement speed of the pitcher's action when batters were required to estimate the speed of an approaching ball. Specifically, a pitching scene was filmed from the batter's perspective, and the pitching movement was edited into a fast or slow movement. In Experiment 1, participants were asked to observe a series of reference pitching movements along with a pitching movement with an altered speed, and to determine which pitch was thrown faster. The ball-flight itself was not presented though and so the ball speed had to be estimated based purely on the pitching movement alone. Results revealed that the batters were more likely to associate faster pitching movements with faster ball speeds (and vice versa), suggesting that the pitching movement speed is information that can be used for estimating the ball speed. In Experiment 2, the participants could see the ball trajectory in addition to the pitching movement. In that case, all ball speeds after release were kept the same, and participants were asked to judge the perceived ball speed as a result of manipulations of the pitching movement speed. Results revealed that the batters perceived the ball speed to be faster when the pitching movement was faster, and slower when the pitching movement was slower, even though the ball speeds were seen and were exactly the same. Further, Takamido et al. found that batters when moving to hit the ball modulated the timing of their hitting movements to correspond to the estimated ball speed (Experiment 3). In other words, batters relied on estimated rather than physical ball information to control their swing [see also, (25–27)]. Thus, the accuracy of the perceptual estimation of the ball speed based on information from both the ball and the pitching movement may make an essential contribution to interceptive skill.

Although Takamido's study has clearly shown the important role of an opponent's movement information for the estimation of ball speed, it is not clear in what manner the two different pieces of information are integrated when making judgements of ball speed. There has been much interest in recent years about how skilled athletes might use and integrate multiple sources of information to make predictive judgements (28, 29). For example, Gray and Cañal-Bruland (30) outlined three sources of information that can be used for interception: situational probability information (e.g., pitch count), movement kinematics (e.g., pitching movement), and ball-flight information (e.g., visible time), and examined how situational and ball-flight information were integrated to guide interceptive actions in a simulated baseball batting task. Specifically, by manipulating the probability that different types of pitches would occur, and the visible time of the ball trajectories, they investigated how this information was integrated when the reliability of the situational probability and ball information varied. Results showed that, consistent with Bayesian integration (31–33), batters could optimally integrate the different sources of information according to the relative reliability of those sources of information [see also, (34, 35)]. That is, the batters in this cue-combination task may have performed a maximum likelihood estimation by optimally combining the different cues in a fashion that gave greater weighting to the information that was more reliable (36). Although the aforementioned study investigated the integration of contextual and ball-trajectory information, this would imply that the changes in perceived ball speed observed by Takamido et al. (24) resulting from changes in the movement speed of the pitcher might also have been caused by a cue-combination process whereby the kinematic and ball-flight information were integrated based on the relative reliability of the two sources. Indeed, in their study, the pitching movement speed did not alter speed judgements in a linear fashion whereby more extreme manipulations increasingly altered the perceived ball speed. Instead, the extreme manipulations of the pitching movement speed (i.e., much faster or slower) had no greater influence on speed judgements than relatively lesser manipulations. Presumably, the reliability of the pitching motion information did not improve the more extreme it became. In that sense, the batters may have been combining the cues in such a way that they were maximizing the integrated likelihood whereby the kinematic (pitching motion) and ball-speed information were integrated optimally according to their relative reliability. The clarification of this will provide new insights into the characteristics of integration between different sources of sensory information.

Although there was some evidence in the Takamido et al. (24) study to suggest that batters integrated information in an optimal manner, only very tentative conclusions could be drawn because batters saw only manipulations of the pitching movement, and no changes in the actual ball speed. If true integration were to occur, then the information offered by the kinematic action should be weighted more heavily if the ball-flight information were to become less reliable. For instance, a batter might rely less on the ball-flight information—and more on the kinematic information—when the ball speed increases. The information about ball speed offered by a faster moving ball may be more difficult to pick-up (e.g., from disparity and relative expansion), and ultimately would result in there being less time available to use the ball information before a making swing decision. Because swing decisions in baseball must be made in an anticipatory manner [e.g., (37)], typically using only information available from the pitcher's kinematics and/or early ball trajectory (2, 3), the reliability of ball information for faster ball speeds is expected to be lower than for slower ones because less information is available before a swing decision is required. This is particularly likely to be the case if the batter has relatively less experience batting against the faster ball speed, in which case the batter would be expected to be less certain about their judgements. Indeed, in previous studies that manipulated reliability by occlusion of the ball-flight trajectory (30, 34), it has been reported that a reduction in visible time decreases the use of ball-flight information.

The aim of this study was to investigate how the perception of ball speed in baseball is modulated in response to changes in the reliability of the pitching movement and ball trajectory. In doing so, we sought to test whether Bayesian-like cue combination would be applied to the perception of ball speed, with the informational cues available from the pitching movement and ball flight being combined optimally according to its reliability (31–33). There were two particular reasons to do so. One is that, as mentioned, we can further our understanding of how skilled players integrate information to achieve highly accurate judgements that support interception (30). Recent evidence suggests that athletic experience may facilitate the use of probabilistic information (i.e., reliability) for optimal sensorimotor estimations (38). The second reason is to progress our understanding of why mismatches occur between the perceived and actual ball-flight information in baseball situations. The mismatch between perception and reality has often been an interesting phenomenon for researchers [e.g., the rising fastball and/or breaking curveball: (1)] and practitioners (e.g., pitchers throwing a ball that feels faster than its physical speed). A better understanding of the manner by which batters integrate ball-flight information may advance the understanding of these phenomena.

**Figure 1:**
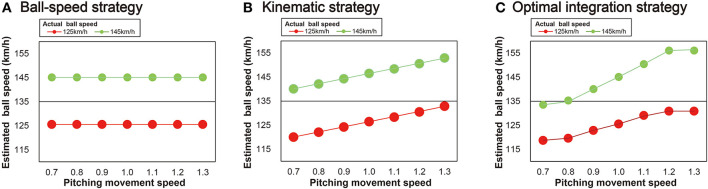
Three possible strategies for ball speed estimation. **(A)** Ball-speed strategy: batters estimate ball speed using only the ball trajectory information. **(B)** Kinematic strategy: batters estimate ball speed using only kinematic information (i.e., the pitching movement speeds) that is unique to each of the two ball-speed conditions (125 or 145 km/h). **(C)** Optimal integration strategy: batters combine both the ball trajectory and pitching movement speed to produce an integrated estimate of ball speed. Note that in **(C)** the information obtained from the ball is considered to be less reliable when the ball speed is higher and so the gradient of the curve in the 145 km/h ball speed condition is greater than it is for the 125 km/h condition (i.e., kinematic information is weighted more heavily). The higher the ball speed, the more weight is given to the pitching movement speed information to estimate the ball speed. Conversely, the farther the pitching movement speed is from the natural speed, the more weight is given to the ball trajectory information to estimate the ball speed.

To achieve our aim, we asked batters to perform a pitch speed-estimation task in a virtual environment in which the reliability of the pitching movement and ball-speed information was systematically manipulated. Specifically, the reliability of the pitching movement was manipulated by modulating the pitching movement speed (24) and the reliability of the ball-flight information was manipulated by modulating the ball speed. When combining information from the pitching movement speed and ball-speed, there appear to be three possible strategies for the batter to estimate ball speed: (1) a ball-speed strategy; (2) a kinematic strategy; and (3) an optimal integration strategy (Figure 1). If using a ball-speed strategy, a batter would estimate the ball speed using only information available from the ball after its release. Modulation of the pitcher's movement speed would not affect the perception of the ball speed (Figure 1A). If using a kinematic strategy, the perception of ball speed would be perceived as a direct function of the movement speed, independent of the ball speed (Figure 1B). If using an optimal integration strategy, the kinematic and ball-speed information would be integrated based on their relative reliability (Figure 1C). According to Bayesian integration, the integration of information should be done in a flexible manner based on the relative reliabilities of the different sources of information. More specifically, when estimating ball speed, we expect more weight to be given to the pitching movement speed information when the ball speed is higher and therefore less reliable. Conversely, more weight should be given to the ball trajectory information the farther the pitching movement speed moves from the natural speed and therefore is less reliable. Based on the findings of the study by Takamido et al. (24), we expected to find that batters integrate the two sources of information in an optimal manner.

## Methods

### Participants

Thirteen right-handed male university level baseball players (mean age = 20.2 ± 0.8 years) with normal or corrected-to-normal vision participated. Participants had been playing baseball for a mean of 11.9 ± 1.7 years and had taken part in regular baseball training and competitions. All participants belonged to the same baseball team though some were starting players (who played most games) and others not. Analysis using G^*^Power (39) indicated that, based on the medium-large effect size (*f* = 0.30) reported in Takamido et al.'s (24) ball-speed evaluation task, a total of 11 participants would be necessary to detect a difference in perceived ball speed with power > 0.80, assuming α = 0.05. All participants were informed of the experimental procedures in advance and consented to participate. The study was approved by the institutional Ethical Review Committee in accordance with the Declaration of Helsinki (no. 22-1-4).

### Visual stimuli and apparatus

We used a custom-made virtual baseball batting environment to present and control the visual stimuli shown during the experimental task. The virtual environment was created using the Unity game engine (Unity Technologies, San Francisco, CA) and consisted of a baseball stadium in which there was a baseball field with a regular-sized pitching mound and batter's box. A baseball was thrown by an avatar toward a pre-specified location (through the center of the home base at a height of 80 cm) at 125 km/h (*slow* condition) or at 145 km/h (*fast* condition). These ball speeds were chosen because the participants in the current study usually faced pitchers who threw their fastball at or around 135 km/h in their regular games. This meant that the 125 and 145 km/h ball speeds were considered to be easier and harder to perceive than their regular speeds, respectively. The ball flight times of the fast- and slow-speed condition were approximately 450 and 530 ms, respectively. Considering that the batter initiates their swing at a particular time before ball arrival, it was assumed that the amount of information was reduced by about 80 ms for the fast- compared to the slow ball-speed condition. This is a similar change in time constraint to the aforementioned occlusion study [50–150 ms: (30)] that manipulated the reliability of ball trajectory information. That is, we assumed that the trajectory of a fast ball-speed condition was a less reliable condition. To reproduce a natural ball trajectory in the virtual environment (using the known release point, arrival point, and ball speed), we calculated a cubic polynomial that best represented the three-dimensional coordinates of a ball at each point in time according to the trajectory calculator created by Nathan (40).

The pitching movement of the avatar was created based on the pitching movement of a professional baseball pitcher when he threw at 135 km/h, captured using a three-dimensional motion capture system (Eagle System, Motion Analysis Corporation, Santa Rosa, CA). The Eagle System incorporated 12 cameras that used a sampling rate of 500 Hz and a shutter speed of 2,000 Hz. The root mean square error in the calculation of the three-dimensional marker location measured during the experiment was <1.0 mm. The same pitching movement was used as the stimulus throughout the experiment irrespective of the ball speed.

An HTC Vive head-mounted display VR system (HTC Corporation, New Taipei City, Taiwan) was used for participants to view the stimuli during the experiment. The system consisted of a headset, a Vive tracker, and two lighthouses that emit infrared laser sweeps to localize the headset and tracker. The HTC Vive has excellent spatial and temporal precision when the participant remains stationary (41, 42). Participants wore the Vive headset to view the virtual environment and attempted to hit the ball using an actual baseball bat that was fitted with a Vive tracker sampling at 90 Hz to determine the position of the bat and to show the bat in the virtual environment.

### Experimental task and procedure

Each participant performed two tasks: (1) a preliminary task to identify the participant-specific “natural” pitching-movement-speed to be used in the experiment, and (2) the experimental task proper.

#### Identification of the participant-specific “natural” pitching-movement-speed

We first sought to establish, for each participant, the “natural” pitching-movement-speed that they perceived to match the 125 and 145 km/h ball speeds. To do so, participants observed scenes in which the movement speed of the avatar was modulated between 0.4 and 1.6 times that of the original avatar movement speed. They did so in two conditions: (1) the “slow” (125 km/h) ball-speed condition; and (2) the “fast” (145 km/h) ball-speed condition. In each trial, participants verbally reported whether the relationship between the pitching movement speed and ball speed felt natural. The presentation order of the 13 pitching movement speeds was arranged in blocks so that they were presented either in an ascending order (from 0.4 to 1.6 times the original speed) or in a descending order (from 1.6 to 0.4 times). Each block was performed five times in each of the slow and fast ball-speed conditions (260 trials in total), for a total of 20 blocks that were presented in a randomized order (2 presentation orders × 2 ball speeds × 5 times). Finally, the pitching movement speed that had the highest probability of being perceived as natural for each of the two ball speeds was used as the “natural” pitching-movement-speed for that participant in the experiment proper. When there were multiple movement speeds with the highest probability of being perceived as natural, those conditions were presented again and the participant was asked to answer which condition felt more natural. This procedure was repeated until the participant selected the stimulus that felt most natural. A comparison of the chosen pitching-movement-speeds for the two ball-speed conditions found that the selected speed for the fast ball-speed condition (*M* = 1.2 times, *SD* = 0.1) was significantly faster than that for the slow ball-speed condition [*M* = 1.1 times, *SD* = 0.1; *t*_(12)_ = −2.38, *p* = 0.03, *r* = 0.57].

Once the natural pitching-movement-speed was established for each ball-speed for each participant, seven versions of the avatar were created for each ball speed to represent pitching movements with different movement speeds. Specifically, seven avatars were created for each ball speed, with movement speeds between 0.7 and 1.3 times (increments step = 0.1 times) the identified natural movement speed for each participant.

#### Experimental task proper

**Figure 2:**
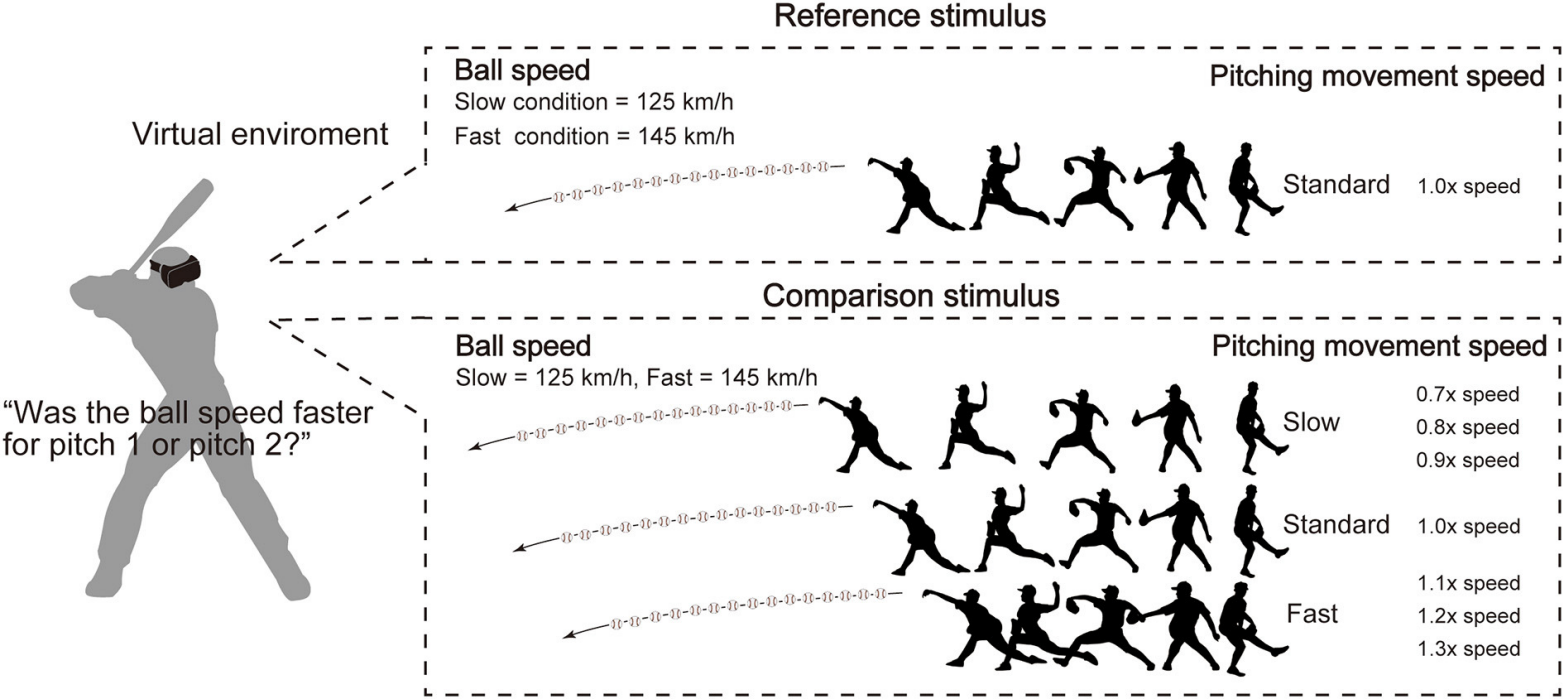
Experimental setup. The task was the ball-speed evaluation task. In each trial, the reference and comparison pitches were presented sequentially in a random order. Participants were asked to verbally report which ball speed was faster. The reference stimulus was presented at the participant-specific natural pitching-movement-speed (i.e., that they perceived to match the ball speed). The comparison stimuli were presented using the same ball speed but using one of seven pre-determined pitching-movement-speeds. The ball speed was always 125 km/h in the slow condition and 145 km/h in the fast condition.

The experimental task was the ball-speed evaluation task used by Takamido et al. (24) (Figure 2). Specifically, in each trial, a reference and comparison pitch were presented sequentially, in a randomized order, each showing a pitching movement along with the subsequent ball-trajectory. Participants were required to swing at each pitch as if they were to hit it, but did not receive visual feedback about whether they did hit it. After attempting to hit the two pitches, participants were asked to verbally report which ball speed they perceived to be faster (i.e., that in the “first” or “second” pitch). Although there has been some criticism of the use of unnatural response modes, including verbal responses, for testing perceptual expertise in interceptive sport (43–45), we used the verbal response because Takamido et al. had already shown that the pitching movement speed influences performance in the ball-speed evaluation task irrespective of whether the participant's response is recorded verbally (24) or through movement (46). The reference stimulus always showed the participant-specific natural pitching-movement-speed. The comparison stimuli were presented using one of the seven predetermined participant-specific movement speeds, including the natural speed. The ball speed was always 125 km/h in the slow condition and 145 km/h in the fast condition.

After warming up with baseball bat-swings performed in the absence of any visual stimulus, participants stood in the batter's box on the virtual baseball field and were instructed on how to respond during the experimental task. Balls were thrown by the avatar from the pitching mound (18.44 m away). Participants started with 28 practice trials that included a set of reference and comparison stimuli for familiarization with the virtual environment and the experimental task (two trials for each of seven movement speeds and two ball-speed conditions, presented in a random order). Participants then performed the experimental task, which included seven blocks of 21 trials for each of the two ball-speed conditions (i.e., 7 pitching movement speeds × 21 repetitions × 2 ball speeds, resulting in a total of 294 trials per participant). The ball-speed conditions were blocked together, with the order of ball-speed conditions counterbalanced across participants. The reason for using a blocked rather than a random order of ball-speed conditions was to avoid contamination of the perceived ball speed of the task by factors other than the pitching movement and ball-speed information (i.e., contextual expectations about the ball speed based on the ball-speed seen on the previous trial). The pitching-movement-speed conditions were presented in a random order within each block of ball speed. Participants were given a short break between blocks and a long break between ball-speed conditions. No feedback about their answer was given to the participants by the experimenter after their responses. The experiments were conducted over 2 days. On the 1st day, participants performed only the preliminary task to identify the “natural” pitching-movement-speed. The other tasks were performed on a 2nd day.

### Data analysis

The probability of trials in which the participant perceived the ball speed of the comparison stimulus to be faster than that of the reference stimulus was calculated per condition for each participant. The probability data were then subject to a 2 (ball speed: slow, fast) × 7 (pitching movement speed: 0.7, 0.8, 0.9, 1.0, 1.1, 1.2, and 1.3 times) repeated measures ANOVA. Degrees of freedom for *F*-ratios were adjusted using the Greenhouse–Geisser procedure if violations of sphericity were encountered. In order to retain the level of statistical power, all *post-hoc* pairwise comparisons were performed using the Shaffer's modified sequentially rejective Bonferroni procedure (47, 48). Effect sizes were estimated using the partial eta-squared measure (η_p_^2^). The 95% confidence interval of the effect size was also calculated. Statistical analyses were conducted using *R* Statistical Software (49). The significance level was set at 5%.

## Results

### How the perception of ball speed is modulated in response to changes in the reliability of the pitching movement and ball trajectory

**Figure 3:**
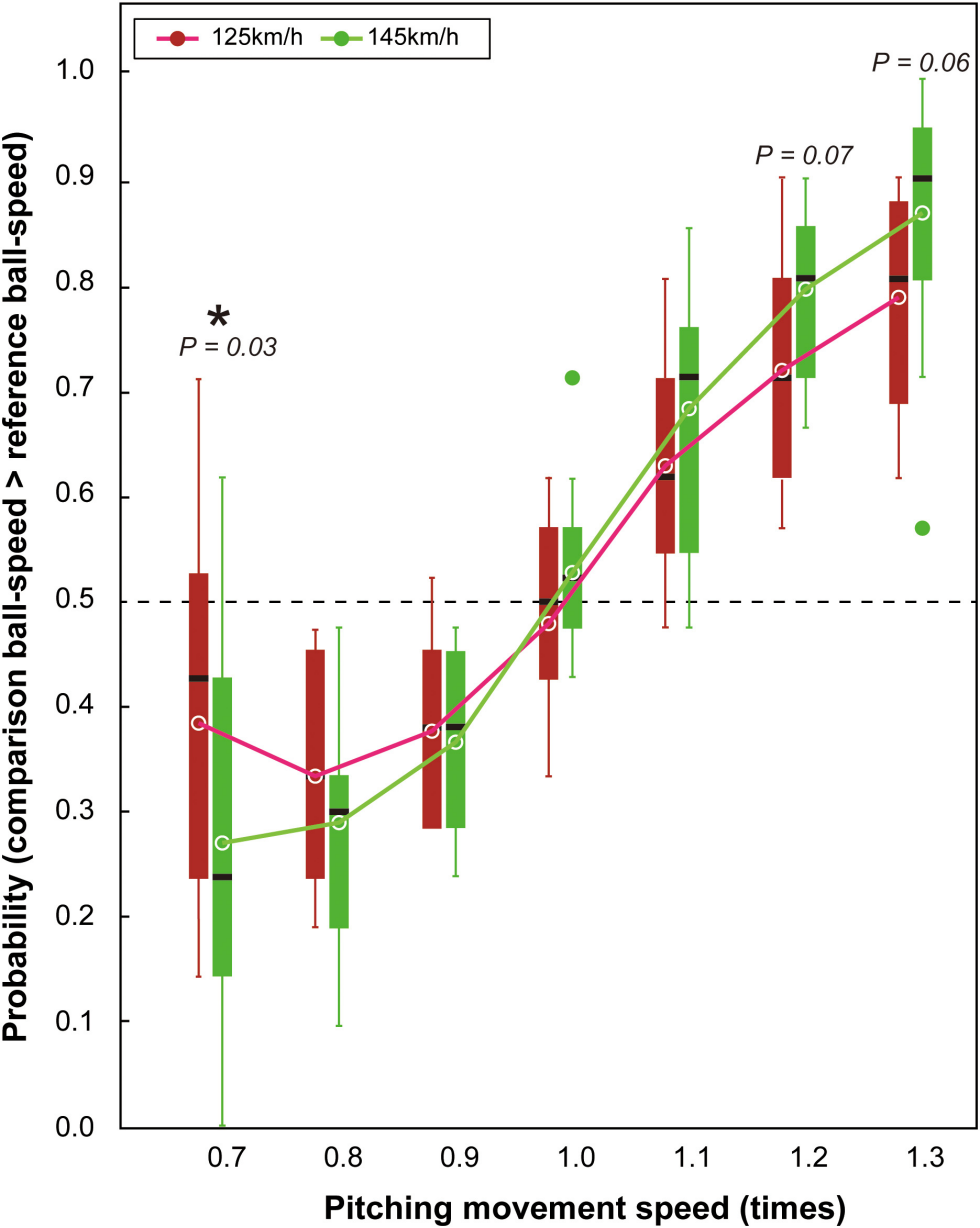
Box plots for probability that participants reported that the comparison ball-speed was faster than the reference ball-speed. The circles shown within the boxes indicate the mean probability in each condition. The horizontal dotted line represents the chance level expected when guessing (50%). The green dots shown outside of the boxes are outliers.

A main effect of pitching-movement-speed [*F*_(2.6,31.2)_ = 72.83, *p* < 0.001, η_p_^2^ = 0.86, 95% CI (0.80–0.89)] revealed that participants perceived the ball speed to be faster when the pitcher's movement speed increased (and vice versa) across both the slow and fast ball-speed conditions (Figure 3). However, as expected, an interaction between the pitching movement speed and ball speed [*F*_(6,72)_ = 3.23, *p* = 0.007, η_p_^2^ = 0.21, 95% CI (0.05–0.38)] showed that the effect of the pitching movement speed on the perceived ball-speed differed between the slow and fast ball-speed conditions. Specifically, the impact of the pitching movement speed on perceived ball-speed was greater in the fast ball-speed condition (145 km/h) than it was in the slow ball-speed condition (125 km/h; see in Figure 3 the greater gradient for the best fit in the fast ball-speed condition). This resulted in a significantly greater effect on perceived ball-speed in the fast ball-speed condition when the pitching movement speed was at its slowest [i.e., when pitching movement speed = 0.7, slow vs. fast: *p* = 0.03, η_p_^2^ = 0.34, 95% CI (0.02–0.64)], and borderline effects when the pitching movement speed was at its fastest [i.e., when pitching movement speed = 1.2, slow vs. fast: *p* = 0.07, η_p_^2^ = 0.25, 95% CI (0.001–0.66); and when pitching movement speed = 1.3, slow vs. fast: *p* = 0.06, η_p_^2^ = 0.26, 95% CI (0.001–0.61)].

To further determine whether the changes in pitching-movement-speed influenced the estimations of ball speed, we conducted chance-level tests for each experimental condition to compare the measured probability in each condition to the 0.5 probability level expected by chance guessing. Findings revealed that the probabilities were significantly different to 0.5 in each of the conditions except when the pitching movement speed was 1.0 times in the 125 km/h condition (*t* = −0.78, *df* = 12, *p* = 0.46), when it was 1.0 times in the 145 km/h condition (*t* = 1.34, *df* = 12, *p* = 0.20), and a borderline effect when the pitching movement speed was 0.7 times in the 125 km/h ball-speed condition (*t* = −2.10, *df* = 12, *p* = 0.06).

### Exploratory analysis

**Figure 4:**
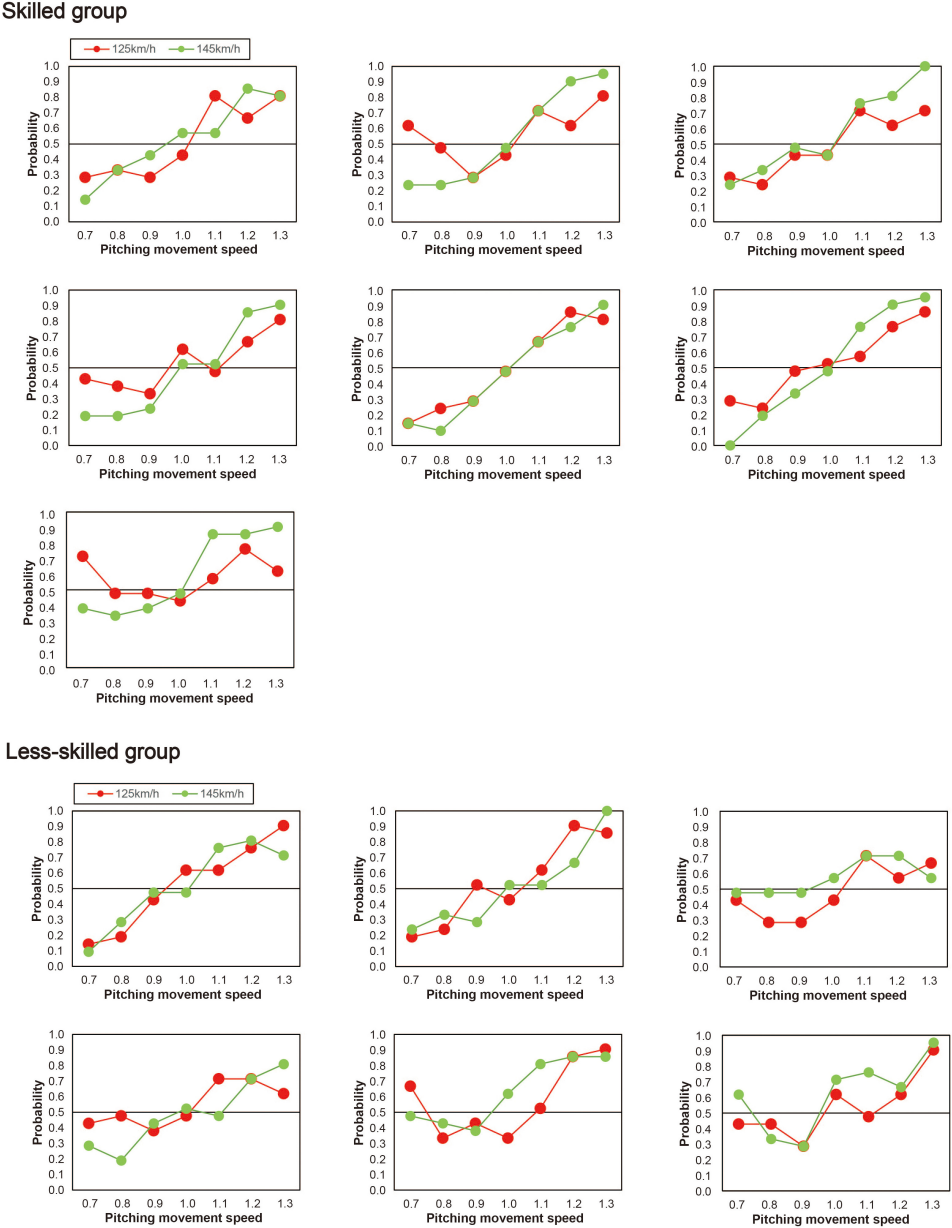
Individual probabilities for the skilled (upper half) and less-skilled participants (lower half). The skilled participants were regulars on their team. The less-skilled group were those who sometimes played in competitive games.

**Figure 5:**
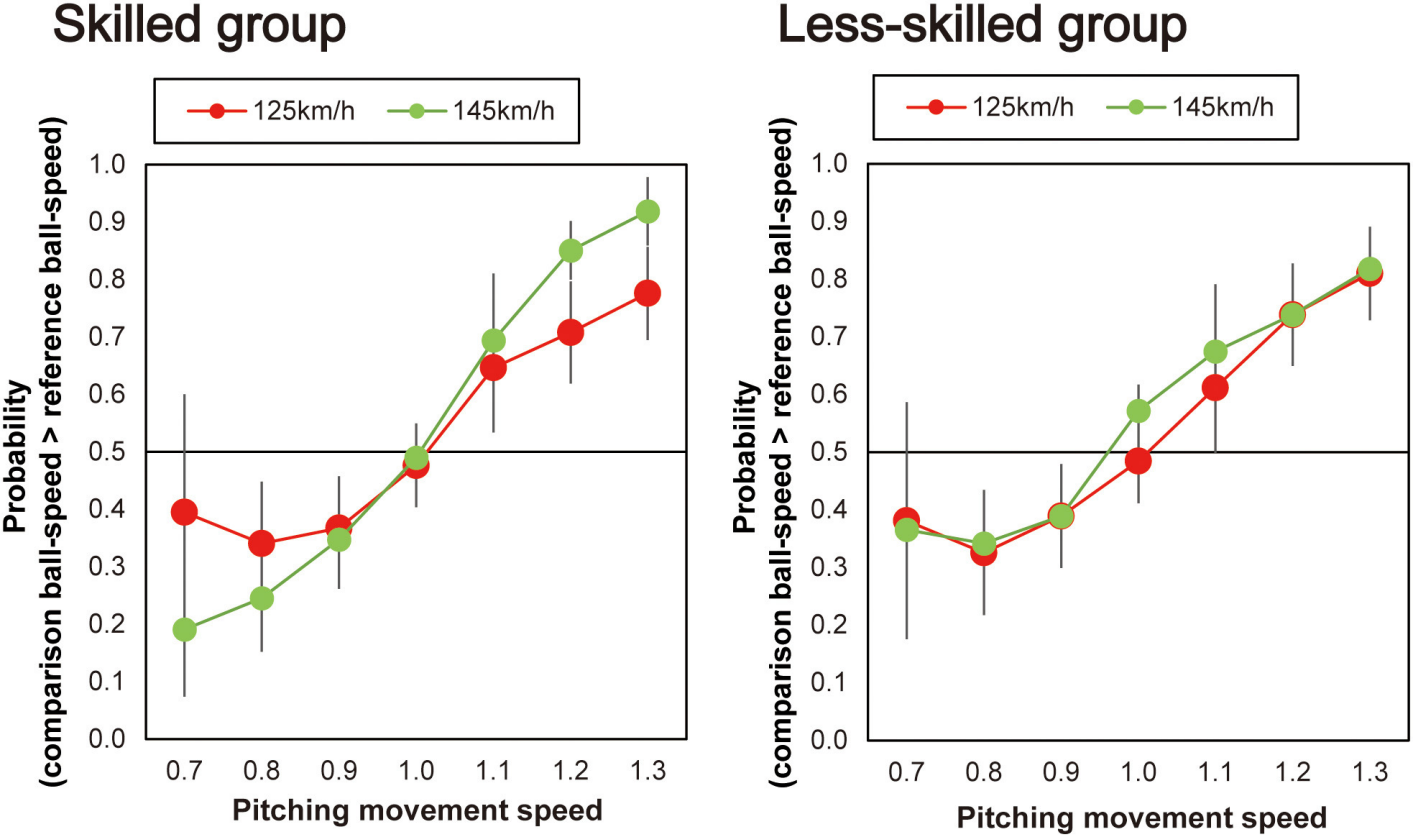
Plots of the mean probability that the skilled and less-skilled participants reported that the comparison ball-speed was faster than the reference ball-speed. The horizontal dotted line represents the chance level expected when guessing (50%).

Next, as shown in Figure 4, individual differences were evident between participants in the way that the pitching movement speed appeared to influence the perceived ball-speed. Specifically, the perceived ball-speed of some participants was systematically influenced by the pitching movement speed more than others, and some were influenced more by the faster ball-speed than others. To explore whether the skill level of the participants influenced this relationship, we divided our participants into two groups based on their skill level (Figure 5). Seven participants (the skilled group) were regular team members who started in all games they played (43.2 ± 2.1 games in a year), and six participants (the less-skilled group) were not regular players, but instead those who occasionally started in the games (3.4 ± 1.1 games in a year). A 2 (group) × 2 (ball speed) × 7 (pitching movement speed) ANOVA revealed a significant three-way interaction [*F*_(6, 66)_ = 2.50, *p* = 0.03, η_p_^2^ = 0.19, 95% CI (0.03–0.29)]. Therefore, we performed a two-way ANOVA for each group and found, in the less-skilled group, a main effect of pitching movement speed [*F*_(6, 30)_ = 21.40, *p* < 0.01, η_p_^2^ = 0.81, 95% CI (0.76–0.86)], but no significant interaction between pitching movement speed and ball speed [*F*_(3.63, 18.16)_ = 0.33, *p* = 0.84, η_p_^2^ = 0.06, 95% CI (0.01–0.09)]. On the other hand, in the skilled group, there was a significant main effect of pitching movement speed [*F*_(6,36)_ = 58.21, *p* < 0.001, η_p_^2^ = 0.91, 95% CI (0.86–0.94)] in addition to a significant interaction between pitching movement speed and ball speed [*F*_(2.59,15.53)_ = 7.31, *p* = 0.003, η_p_^2^ = 0.55, 95% CI (0.28–0.73)]. Simple main effects for the skilled group indicated that the impact of the pitching movement speed on perceived ball-speed was significantly greater in the fast ball-speed condition when the pitching movement speed was slowest [i.e., when pitching movement speed = 0.7, slow vs. fast: *p* = 0.01, η_p_^2^ = 0.71, 95% CI (0.33–0.93)], and tended to be greater when the pitching movement speed was 0.8 times [*p* = 0.07, η_p_^2^ = 0.45, 95% CI (0.001–0.84)]. Similarly, the impact of the pitching movement speed was significantly greater in the fast ball-speed condition in the 1.2 and 1.3 times pitching-movement-speed conditions [1.2 times: *p* = 0.02, η_p_^2^ = 0.62, 95% CI (0.004–0.95); 1.3 times: (*p* = 0.01, η_p_^2^ = 0.66, 95% CI (0.22–0.79))]. There was no difference in the natural pitching-movement-speed between the skilled and less-skilled groups [*F*_(1,11)_ = 1.29, *p* = 0.28, η_p_^2^ = 0.11], nor any interaction between the skill of the groups and the ball-speed condition [*F*_(1,11)_ = 0.42, *p* = 0.53, η_p_^2^ = 0.04].

Given the exploratory nature of the way that we split our participants into a “skilled” and “less-skilled” group, we ran permutation testing to check how likely it would have been to find the three-way group × ball speed × pitching movement speed interaction if the participants were randomly split into two groups. To do so, we randomly allocated seven of the 13 participants to a first group and the remaining six to a second group. We then ran the ANOVA to determine the *p*-value for the three-way interaction. We ran 10,000 bootstrapped samples of this random allocation to establish the probability that we would find a significant three-way interaction. The results revealed that the probability of finding a three-way interaction when splitting the groups arbitrarily was *p* = 0.049 (i.e., only 487 of the 10,000 samples resulted in a significant three-way interaction). These results provide compelling evidence for the idea that the three-way interaction found when comparing the skilled and less-skilled groups was likely to be a genuine rather than a spurious effect.

## Discussion

The aim of this study was to examine the degree to which kinematic and ball-flight information are integrated when estimating ball speed in baseball batting. Thirteen university level baseball batters performed a ball-speed evaluation task where they were required to determine which of two comparison baseball pitches they perceived to be faster. We manipulated the replay speed of the pitching movement, and the ball speed itself, to determine the degree to which the estimation of ball speed would be influenced by the kinematic and ball-flight information. Consistent with the optimal integration strategy hypothesis, results revealed that the perceived ball speed was clearly influenced by the pitching movement speed, and by the ball speed, with a faster ball-speed being more influenced by the manipulation of the pitching movement. In addition, the pitching movement speed did not alter speed judgements in a linear fashion. Instead, the extreme manipulations of the pitching movement speed (i.e., much faster or slower) had a relatively comparable influence on the speed judgements as the less extreme manipulations. Moreover, exploratory analyses suggest that the more skilled batters were more likely to integrate the two sources of information according to their relative reliability when making judgements of ball speed. The results provide important insights into how skilled performers may make judgements of speed and time to contact, and further enhance our understanding of how the ability to make those judgements might improve when developing expertise in hitting.

Takamido et al. (24, 46) previously demonstrated that advance kinematic information is integrated with ball trajectory information when batters make judgements of ball speed, and we were able to replicate that finding in our study using a virtual reality paradigm. Participants in the studies by Takamido et al. viewed a video projection showing the pitching movement and subsequent ball-flight information available when viewing a softball pitch and were required to make perceptual judgements about the ball speed (24), or to produce a hitting movement as if to hit the ball seen on the video projection (24, 46). Irrespective of the response type, the speed of the pitching movement altered the participants' perception of ball speed. We were able to replicate this finding in our study, with participants judging ball-speeds to be faster when the pitching movement speed increased, and slower when the pitching movement speed decreased. We also found that the effect of the modulation of the pitching movement speed on the judgment of ball speed becomes smaller when the modulation of the pitching movement speed is extreme. In this task, where the physical speed of the ball is constant between the reference and comparison stimuli, it could have been possible for the participants to respond based solely on the pitching movement speed. However, this does not appear to have been the case. In particular, participant responses did not always align with that expected on the basis of the pitching movement speed, even when viewing the most extreme manipulations of the pitching motion (i.e., the 0.7x and 1.3x speed conditions; see Figure 3). In contrast, in Takamido's study, when only the pitching movement was presented (i.e., without the ball trajectory) and the participants were asked to judge the ball speed, participants responded accurately almost all of the time. Accordingly, it appears that the decisions of the participants in our study were indeed influenced at least in part by the ball speed itself. Our replication of Takamido et al.'s findings is not a trivial finding given that we used a computer-generated avatar of a pitcher (and the subsequent ball-flight trajectory) rather than video footage of an actual pitch as they did. The ability to examine task performance in a virtual environment allows the presentation of three-dimensional trajectories that are known to provide important cues for time-to-contact in hitting (10, 12–14), and are likely to be perceived as more realistic than the two-dimensional trajectories seen when viewing video projections (50, 51). Moreover, virtual environments offer the opportunity to examine the impact of manipulations in pitching movements and ball trajectories that might not otherwise be possible using video projections (50, 52).

Crucially, in our study we were able to extend the findings of Takamido et al. (24, 46) by showing that the integration of the advance kinematic and ball-flight information is influenced by the speed of the ball. We used two different ball speeds and hypothesized that, if we presented ball speeds that were above and below the threshold ball-speed typically experienced by our participants during regular games, then the influence of the pitching movement speed would be greater in the fast ball-speed condition than it would in the slow ball-speed condition. The rationale for this choice was that the participants would be less certain about their judgements of the faster ball-speed and therefore would—according to Bayesian integration—rely more in the fast ball-speed condition on the information available from the pitching movement. Consistent with our hypothesis, the degree to which the judgements of ball speed were influenced by the pitching movement speed was greater for the fast ball-speed than they were in the slow ball-speed condition (see Figure 3). These findings suggest that baseball batters integrate information about ball trajectory in an optimal “Bayesian-like” manner whereby the influence of each source of information is altered according to its (un)certainty. However, the relationship between pitching movement speed and perceived ball speed was not a linear one. Instead, there appears to be a limit to which the alteration of the pitching movement speed will influence the judgements of ball speed (e.g., see the flattening of the influence of the slowest pitching movement speeds in Figure 3). These findings add to the growing body of evidence which shows that perceptual judgements in sport are produced by integrating different sources of information in an optimal manner according to the degree to which that information can be relied on (28, 30, 34, 35).

Our exploratory analysis provided some evidence that a relationship might exist between skill in batting and the degree to which information is integrated when making judgements of ball trajectory. When we divided our participants into a skilled and less-skilled group on the basis of their contribution to their university team, we found that only the skilled group used the ball-speed information to modulate the way that they integrated the kinematic and ball-speed information. When making their judgements of ball speed, the skilled players were more likely to rely on the pitching movement speed in the fast ball-speed condition (145 km/h) than they were in the slow ball-speed condition (125 km/h). It should be noted though that this interpretation relies in some cases on borderline effects (i.e., when comparing integration of the skilled participants when the pitching movement speeds were at their fastest; see Figure 5). Nonetheless, the skilled players appeared to act in a more Bayes-optimal manner in that they relied less on the kinematic information when the ball-speed was presumably more reliable (and indeed a speed they were more accustomed to), and more on the kinematic information when the ball speed was less reliable. Indeed while it is known that individuals rely more on kinematic information when time constraints are higher, it has remained unclear how the multiple sources of information are combined to make perceptual judgements. Bayesian integration helps us understand how the integration might occur, that is, by weighting the multiple sources of information according to their respective reliability. In contrast, the judgements of the less-skilled players did not differ according to ball speed. Pitching movement speed influenced the estimations of ball speed in the same manner irrespective of the actual ball speed (i.e., 125 or 145 km/h, see Figure 5). Although only so far based on a preliminary analysis, this result provides a suggestion that more skilled players may have a better capacity to integrate difference sources of information when making judgements of ball speed [for an analogous finding in Musicians, see (53)]. This may provide the more skilled batters with a better capacity to, for instance, better adjust to faster ball speeds in the natural environment where the pitching movement speed would be expected to more closely align with the actual ball speed in a more natural fashion. If true, this finding would represent an important advance in our understanding of the nature of the expert advantage in hitting [see also, (34)].

As mentioned in the introduction, in a real batting situation, players sometimes mention that pitchers throw a ball that feels faster than the actual ball speed. The results of this study suggest a possible mechanism: that those pitchers may be those with a fast pitching-movement-speed. On the other hand, batters also mention that ball flight feels faster than the physical ball speed when they are faced with a pitcher who uses a slower pitching motion or who uses less apparent effort in pitching. At first glance, this phenomenon appears to contradict the results of this study in finding that batters tended to perceive a slower ball speed as the pitcher's movement speed decreased. The present results and previous study, however, can be used to explain the phenomenon. Specifically, in addition to the present finding that the batter's judgement of ball speed modulates the speed of the pitching motion, Takamido et al. (24), who recorded actual swing movements, found delayed responses occurred when a slow ball was perceived. The important point here is that in the above case (i.e., perceived slower), the batter underestimates the physical ball speed and then the batter actually produces a delayed response. In general, when batters did make a delayed response, they would think the ball speed was faster than expected. In other words, the perception of a ball speed that is slower than the physical ball speed results in the feeling that the actual ball was faster. This interpretation does not contradict the intuition in the field, and the results of this study, but rather may explain why there is a gap between what occurs and what is perceived in practice.

A key consideration when evaluating the implications of this study is to evaluate the degree to which the experimental manipulations of pitching movement speed and ball speed can be considered to be manipulations of “reliability.” Bayesian integration states that the relative contribution of different sources of information should be weighted according to the reliability of that information (31–33). In this study, we hypothesized that the manipulation of pitching movement speed away from its “natural” speed would represent a manipulation of the reliability of that information, and that the ball-speed would be less reliable if increased to a speed above that which batters would be typically accustomed to. In the end, the findings aligned with our predictions: the additional contribution of the information available from the kinematic pitching movement diminished when at its extremes of manipulation (24, 46), and the ball-speed was used less when the speed increased (at least it did for the skilled batters). Nonetheless, it could be that these outcomes are found not because the information is more “reliable,” but instead because it becomes less feasible/realistic (in the case of the pitching movement) and/or because participants were less accustomed to some of the speeds (for both the pitching movement and ball speed). In other words, the method of indirectly manipulating reliability by manipulating the content of information, as we have done in this study, has also varied the content of the information available to the batters [as it also would have if we had, for instance, used visual occlusion to alter the reliability of the information, e.g., (30)]. To further test the idea that judgements are made by weighting information according to its reliability, other manipulations of pitching movement and ball speed may be used as alternative means of altering reliability. For instance, the pitching movement could be made less reliable by introducing masking and/or random noise to a point light display or by manipulating the exaggeration of the kinematics [e.g., see (35)]. The reliability of the ball-speed information could be manipulated by blurring the information [e.g., (44, 54)] or by occluding portions of the ball flight (30).

A further consideration relates to the degree to which a verbal judgement of ball speed can be considered to be related to the actual judgements made when batting at the plate in an actual game. We based our performance measures in this study on the verbal responses of our participants rather than using a movement-based response (though participants were asked to try hit each pitch in our study). This decision was made in large part because Takamido et al. had already shown that the pitching movement speed influences performance in the ball-speed estimation task irrespective of whether the participant's response is recorded verbally (24) or through movement (46). Nonetheless, it has been shown that a verbal or even simplified movement response can under-represent the true nature of the expert advantage when tested using a movement response relied on in the natural environment [e.g., see (44)]. Accordingly, even stronger effects might be expected if the study were to be replicated by testing a movement rather than verbal response. It seems necessary, however, to study not only perceptual responses but also motor responses, since some salient features that can be observed in motor responses may not appear in perceptual responses, as exemplified by research built on the two visual stream hypothesis and/or on representative design [see (55) for a recent discussion in sport].

In this experiment we asked participants to make explicit judgements about ball speed, and it is possible that the confidence that the participants had in their ability to make those judgements may have influenced their results. In particular, the skilled baseballers might have had more confidence in their ability and this could have aided their apparent advantage in the task. A close relationship exists between perceptual judgements and our metacognitive ability to express confidence in those visual judgements (56, 57). In particular, confidence itself can control perceptual judgements when knowledge is accumulated over time (58). Ota et al. (59–61) have even shown that people tend to become overconfident and that this can lead to sub-optimal integration when combining different sources of information. However, skilled athletes are known to have developed strategies to generally optimize the way that they integrate sensory information (35) and it could be that skilled athletes have developed strategies to overcome these tendencies. Studies of anticipation and decision making in sport have at times incorporated measures of confidence [e.g., (62)], though typically to make inferences about the degree to which participants might have explicit (or implicit) awareness of their knowledge on which they base their decisions. It would be interesting for future studies to assess confidence when making first order judgements of the type made in this study to try and disentangle the role that confidence might have in facilitating (or even inhibiting) the decision making of skilled athletes.

As described above, this study was able to clarify the perceptual characteristics of batters by using a virtual reality environment that allows flexible manipulation of the information sources. It should be noted, however, that the findings in such a manipulated environment might not fully reflect those likely to be found in a real-life scenario. For example, in this study, we verified the perceptual characteristics of batters by mechanically changing the overall speed of a pitching movement, in accordance with previous research (24, 46). However, in real-life situations, when a pitcher manipulates the ball speed, changes to only parts of that movement (e.g., the arm speed) would be necessary to alter the overall ball speed. It has been reported that there is a relationship between the angular velocity of specific body parts and the ball speed in baseball pitching (63–65), but that this relationship is not a simple linear one. Therefore, maintaining the naturalness of real world while taking advantage of the manipulability of virtual environment will progress the research of perceptual expertise.

In sum, the results of this study show that baseball batters integrate multiple sources of information when making judgements of ball speed. Specifically, batters in our study integrated kinematic (pitching movement) information and information about the ball trajectory to make judgements of the speed of the approaching ball. Exploratory analyses provided some suggestion that the skilled batters within our relatively homogenous group were able to more flexibly integrate the two sources of information according to its presumed reliability, in particular, by greater weighting the pitching movement information when the ball speed increased to a speed that they were less accustomed to. If true, this may provide skilled batters with a functional adaptation to adjust to and anticipate the arrival time of the ball when faced with the more severe time constraints inherent with faster ball speeds.

## Data availability statement

The raw data supporting the conclusions of this article will be made available by the authors, without undue reservation.

## Ethics statement

The studies involving human participants were reviewed and approved by National Institute of Fitness and Sports in Kanoya. The patients/participants provided their written informed consent to participate in this study.

## Author contributions

HN, KF, and TT designed and performed the experiments. HN built the VR system. HN, DM, and TT analyzed the data and drafted the manuscript. HN, KF, TT, RT, and DM discussed the results and reviewed the manuscript. All authors approved the submitted version.

## Funding

This research was supported by grants-in-aid for scientific research from the JSPS (No. 19H04001) to HN.

## Conflict of interest

The authors declare that the research was conducted in the absence of any commercial or financial relationships that could be construed as a potential conflict of interest.

## Publisher's note

All claims expressed in this article are solely those of the authors and do not necessarily represent those of their affiliated organizations, or those of the publisher, the editors and the reviewers. Any product that may be evaluated in this article, or claim that may be made by its manufacturer, is not guaranteed or endorsed by the publisher.
